# Contrasting patterns of longitudinal population dynamics and antimicrobial resistance mechanisms in two priority bacterial pathogens over 7 years in a single center

**DOI:** 10.1186/s13059-019-1785-1

**Published:** 2019-09-02

**Authors:** Matthew J. Ellington, Eva Heinz, Alexander M. Wailan, Matthew J. Dorman, Marcus de Goffau, Amy K. Cain, Sonal P. Henson, Nicholas Gleadall, Christine J. Boinett, Gordon Dougan, Nicholas M. Brown, Neil Woodford, Julian Parkhill, M. Estée Török, Sharon J. Peacock, Nicholas R. Thomson

**Affiliations:** 1 Public Health England, National Infection Service, Addenbrooke’s Hospital, Hills Road, Cambridge, CB2 0QW UK; 2 Wellcome Sanger Institute, Wellcome Genome Campus, Hinxton, Cambridge, CB10 1SA UK; 3 Antimicrobial Resistance and Healthcare Associated Infections (AMRHAI) Reference Unit, National Infection Service, Public Health England, 61 Colindale Avenue, London, NW9 5EQ UK; 4 Department of Vector Biology, Liverpool School of Tropical Medicine, Pembroke Place, Liverpool, L3 5QA UK; 5 Department of Molecular Sciences, Macquarie University, Sydney, 2109 Australia; 6 KEMRI-Wellcome Trust Research Programme, CGMRC, Kilifi, Kenya; 7 Department of Medicine, University of Cambridge, Addenbrooke’s Hospital, Hills Road, Cambridge, CB2 0QW UK; 8 Cambridge University Hospitals NHS Foundation Trust, Hills Road, Cambridge, CB2 0QQ UK; 9 London School of Hygiene and Tropical Medicine, Keppel Street, London, WC1E 7HT UK; 10 Present address: National Infection Service, Public Health England, 61 Colindale Avenue, London, NW9 5EQ UK

**Keywords:** Resistance mechanisms, Population dynamics, Intrinsic resistance, Plasmid diversity

## Abstract

**Background:**

Two of the most important pathogens contributing to the global rise in antimicrobial resistance (AMR) are *Klebsiella pneumoniae* and *Enterobacter cloacae*. Despite this, most of our knowledge about the changing patterns of disease caused by these two pathogens is based on studies with limited timeframes that provide few insights into their population dynamics or the dynamics in AMR elements that they can carry.

**Results:**

We investigate the population dynamics of two priority AMR pathogens over 7 years between 2007 and 2012 in a major UK hospital, spanning changes made to UK national antimicrobial prescribing policy in 2007. Between 2006 and 2012, *K. pneumoniae* showed epidemiological cycles of multi-drug-resistant (MDR) lineages being replaced approximately every 2 years. This contrasted *E. cloacae* where there was no temporally changing pattern, but a continuous presence of the mixed population.

**Conclusions:**

The differing patterns of clonal replacement and acquisition of mobile elements shows that the flux in the *K. pneumoniae* population was linked to the introduction of globally recognized MDR clones carrying drug resistance markers on mobile elements. However, *E. cloacae* carries a chromosomally encoded *ampC* conferring resistance to front-line treatments and shows that MDR plasmid acquisition in *E. cloacae* was not indicative of success in the hospital. This led to markedly different dynamics in the AMR populations of these two pathogens and shows that the mechanism of the resistance and its location in the genome or mobile elements is crucial to predict population dynamics of opportunistic pathogens in clinical settings.

**Electronic supplementary material:**

The online version of this article (10.1186/s13059-019-1785-1) contains supplementary material, which is available to authorized users.

## Background

Since the early 2000s, successive pandemics of bacterial resistance, first- to third-generation cephalosporins and then to carbapenems, have eroded the utility of our most useful antimicrobials (the beta-lactams). *Klebsiella pneumoniae* and *Enterobacter cloacae* are amongst the primary threats to human health due to the range of hospital-acquired infections (HAIs) that they can cause and their potential for becoming multidrug-resistant [1]. Through the increase of antimicrobial resistance, considerable research has focused on understanding opportunistic pathogens and especially the different mechanisms conferring AMR.

The *K. pneumoniae* genome encodes for an ancestrally acquired penicillinase leaving it readily treatable with cephalosporins and other antimicrobials. The widespread acquisition of mobile CTX-M and other extended-spectrum beta-lactamases (ESBLs) encoded on conjugative (transferable) plasmids [2, 3] has been a major driver in the success of *K. pneumoniae* as hospital pathogen. *E. cloacae* carry a chromosomally encoded AmpC beta-lactamase which has a wide spectrum of activity conferring intrinsic resistance to many front-line treatment options including penicillins, penicillin-penicillinase inhibitor combinations, and (when *ampC* becomes derepressed) third-generation cephalosporins, making it a major contributor towards intrinsic resistance for *Enterobacter* spp.

Both *K. pneumoniae* and *E. cloacae* remain largely susceptible to other important groups of antimicrobials in the UK [4]. This includes key drugs such as fluoroquinolones, where resistance can derive from acquired genes but is often caused by chromosomal mutations of the enzymes encoded by *gyrA* and *parC*. Other examples of gene acquisitions driving resistance include the aminoglycosides [5, 6] and other antimicrobials such as chloramphenicol or tetracycline, whereby a single resistance cassette or plasmid acquisition can render a bacterium resistant against several classes of antimicrobials with a single acquisition event.

The *K. pneumoniae* species complex is highly diverse, with three different species predominating amongst human, environmental, and animal isolates [7]. *K. pneumoniae* comprises four main lineages: *K. pneumoniae* subsp. *pneumoniae*; *K. quasipneumoniae* subsp. *quasipneumoniae* and subsp. *similipneumoniae*; and *K*. *variicola.* The vast majority of hospital isolates belong to *K. pneumoniae* subsp. *pneumoniae* (*K. pneumoniae* sensu stricto). From whole-genome sequencing studies comparing outbreaks of drug-resistant *K. pneumoniae* in both well-resourced and poorly resourced healthcare settings [8, 9], it is clear that there are successful high-risk clones that re-occur in independent settings globally [10]. In common with *K. pneumoniae, E. cloacae* comprises a diverse species complex with recent reclassification and revision into 18 *E. cloacae* subspecies clusters [11].

Whilst it is appreciated that both species are highly diverse and multiple lineages and/or clones cause significant health burden, it has become clear that we lack a basic understanding of their population dynamics in clinical settings. Most studies focus on short time frames and provide only short glimpses into the population structure. This significantly limits our understanding of the change in diversity over time and the dynamic gain and loss of antimicrobial resistance genes/mutations over time. To address this, we undertook a 7-year longitudinal study to gain better resolution of the temporal flux of AMR at the Cambridge University Hospitals NHS Foundation Trust (CUH), a 1000-bed secondary and tertiary referral hospital in the UK. We examined the relative contributions of the bacterial lineage itself and the mobile AMR determinants towards the temporal flux in the phenotypic patterns of AMR seen in this setting. To provide the context for the changing patterns of disease and AMR in CUH, we relate these data to national AMR surveillance data and global genomic data for these pathogens. We show that *K. pneumoniae* lineages were successively replaced by new lineages entering CUH that had acquired ESBLs, such as *bla*_CTX-M-15,_ and were disseminated internationally. *E. cloacae* isolates on the other hand showed little temporal flux and were highly diverse, largely unlinked to international spread and reliant on intrinsic cephalosporin resistance via the endogenous AmpC enzyme for AMR. Our study highlights the need for more mechanistic pathogen-specific approaches to control antimicrobial resistance and antimicrobial-resistant bacteria.

## Results

### Longitudinal patterns of antimicrobial resistance in *K. pneumoniae* and *E. cloacae*

**Fig. 1:**
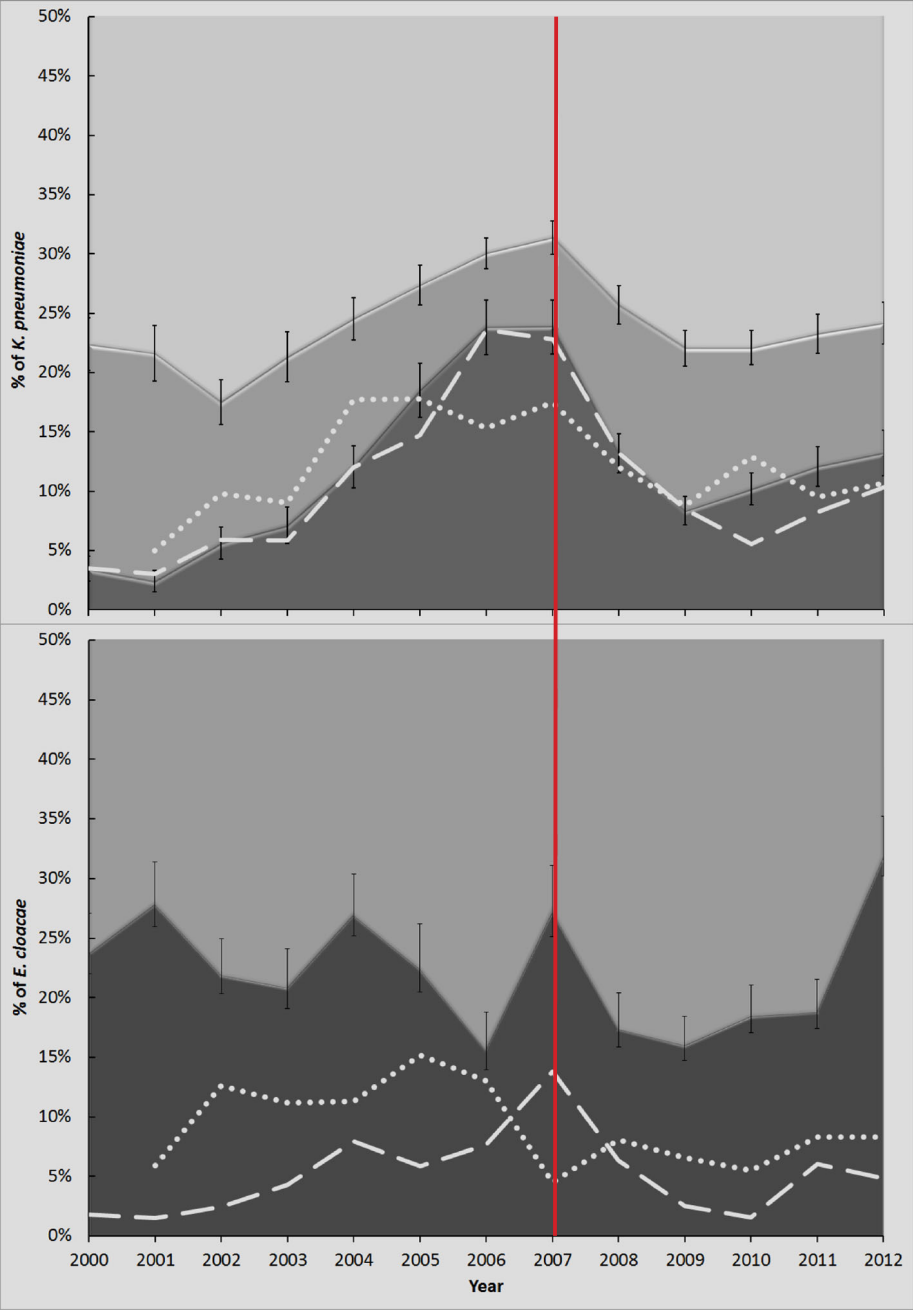
Trends in drug resistance amongst *K. pneumoniae* and *Enterobacter* spp*.* isolated from patients attending CUH between 2000 and 2012. The proportion of **a** *Klebsiella pneumoniae* and **b** *Enterobacter* spp*.* resistant to one group of antimicrobials or less (pale shading), two groups (mid-shading), or three or more groups (dark shading) are shown, and a major change in national antimicrobial prescribing practice in 2007 is denoted with a red vertical line. Black vertical lines represent 95% confidence intervals. Resistance to cephalosporins and/or aminoglycosides in *K. pneumoniae* or aminoglycosides in *E. cloacae* is shown for isolates from Cambridge alone (dashed white line) and the UK (dotted white lines) (UK data from www.bsacsurv.org)

Patient and isolate metadata were collected for the first-recorded *K. pneumoniae* isolate cultured from 13,379 patients and the first *E. cloacae* from 5661 patients attending CUH between 2001 and 2012 (summarized in Fig. 1). As such, patients at CUH were 2.4 times more likely to have a positive *K. pneumoniae* culture compared to *E. cloacae* during this period*.* For both species complexes, the sample types were mainly blood, followed by urine, wounds, and sputum, although *E. cloacae* were isolated from a wider range of body sites (arm/leg, groin, breast, pelvic) compared to *K. pneumoniae* (Additional file 1: Figure S1).

Antimicrobial resistance data showed that *K. pneumoniae* isolates had reduced susceptibility to penicillins (as expected for the species), whilst 6–19% of isolates in a year were resistant to two of the six antimicrobial groups tested (see the “Materials and methods” section; Fig. 1a). The proportion of *K. pneumoniae* isolates with phenotypic resistance to three or more groups varied considerably over time (3.5 to 24%), with peaks in resistance occurring in 2006–2007 and 2011–2012 (Fig. 1a). These peaks were associated with flux in the proportion of isolates resistant to third-generation cephalosporins or aminoglycosides (Fig. 1a). For *E. cloacae*, the background proportions of antimicrobial resistance were higher*,* with 66–82% of isolates resistant to at least two of the antimicrobial groups tested (Fig. 1b). Unlike *K. pneumoniae,* multiple peaks in the proportion of isolates resistant to one or more additional groups were seen more often, in 2001, 2004, 2007, and 2012 (Fig. 1b).

The differing trends in proportions of resistant *K. pneumoniae* and *E. cloacae* at CUH mirrored trends in national surveillance data during the same time period (71) (Fig. 1). These all had a transient downward trajectory immediately after a change in 2007 to national antimicrobial prescribing policy that aimed to decrease cephalosporin usage [12].

### *K. pneumoniae* and *E. cloacae* at CUH between 2006 and 2012 reflected the wider diversity of the species

To investigate the basis of these different trends between the species, we selected 162 *K. pneumoniae* and 132 *E. cloacae* isolates from 158 and 132 patients, respectively, for sequencing. Isolates were collected between 2006 and 2012 from blood and a range of other body sites (Additional file 1: Figure S1), representing 20–34% of the total invasive *K. pneumoniae* and 40–45% of invasive *E. cloacae* isolated at CUH, in any year between 2006 and 2012. The isolates included 114 and 92 isolates that constituted all of the invasive isolates that were resistant to three or more of the six antimicrobial groups tested for *K. pneumoniae* and *E. cloacae,* respectively, between 2006 and 2012*.* Since there were no fully susceptible isolates collected during this period, 48 *K. pneumoniae* and 40 *E. cloacae* isolates resistant to no more than two antimicrobial groups were selected at random for inclusion as a (*c*. 10%) sub-sample of “susceptible” comparators (Table 1).

**Table 1:** *K. pneumoniae* and *E. cloacae* collected in 2006–2012 that underwent whole-genome sequencing

Resistance to drug groups	*K. pneumoniae* isolates (*n*)			*E. cloacae* isolates (*n*)		
	Available (2006–2012)	Sequenced		Available (2006–2012)	Sequenced	
		2006–2012	Q4 2012		2006–2012	Q4 2012
0	0	0	0	0	0	0
1	279	28	3	24	12	3
2	104	15	2	128	16	9
3	4	4	4	50	44	8
4	29	28	8	2	2	3
5	6	6	5	19	19	14
6	43	43	16	2	2	1
Total	465	124	38	225	95	38

Isolates were stratified according to the number of antimicrobial groups to which they were phenotypically resistant by routine diagnostic testing

**Fig. 2:**
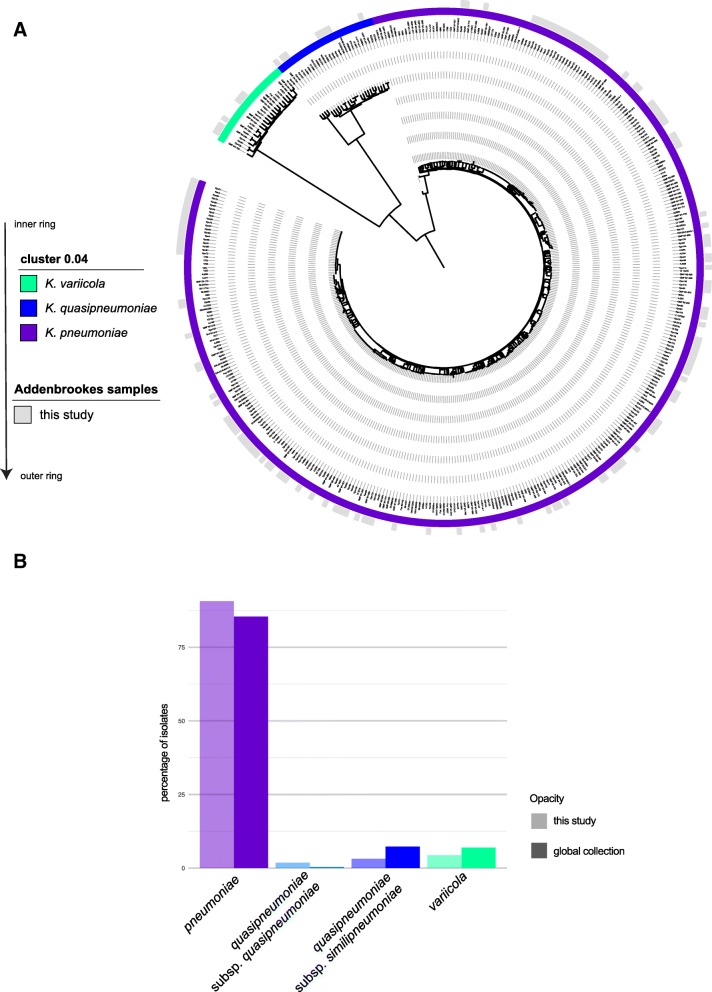
*K. pneumoniae* isolates from CUH within the global population structure. **a** Phylogenetic tree reconstruction of the core gene alignment again highlights the diversity encountered in routine hospital samples. The inner ring shows mash clusters [13] which split the three species *K. pneumoniae*, *K. quasipneumoniae*, and *K. variicola*, at a cutoff of 0.04 correlating to an ANI of 96%. The outer ring displays which samples are derived from this study, embedded in the global diversity [7]. **b** Barplot illustrating the numbers of the different lineages observed in our collection compared to the global collection [7]

Although all isolates had been classified by the routine microbiology laboratory as *K. pneumoniae*, the whole genome phylogeny of *K. pneumoniae* revealed other species within the species complex*;* 15 isolates were *K. quasipneumoniae* or *K. variicola* (Fig. 2a; Additional file 1: Figure S2A; Additional file 2: Table S1). Genotypic resistance predictions indicated that at CUH, the other species were not predominated by multi-drug-resistant isolates (Additional file 1: Figure S3), emphasizing that by contrast, *K. pneumoniae* sensu stricto were the subspecies comprising the majority of multidrug-resistant isolates.

When combined with a global collection representing the *K. pneumoniae* population structure [7] (Fig. 2a, Additional file 2: Table S2), our phylogeny showed that the diversity seen at CUH was representative of the global population diversity, with all major lineages represented. This included the relatively rare subspecies: *K. q.* subsp. *quasipneumoniae*, *K. q.* subsp. *similipneumoniae*, and *K. variicola* (Fig. 2b). Comparison with a UK collection sampled during a similar time frame [14] (Additional file 1: Figure S2A; Additional file 2: Table S3) showed that the CUH *K. pneumoniae* reflected the contemporaneous national distributions across the species complex and the diversity of sequence types (Additional file 1: Figure S2A).

**Fig. 3:**
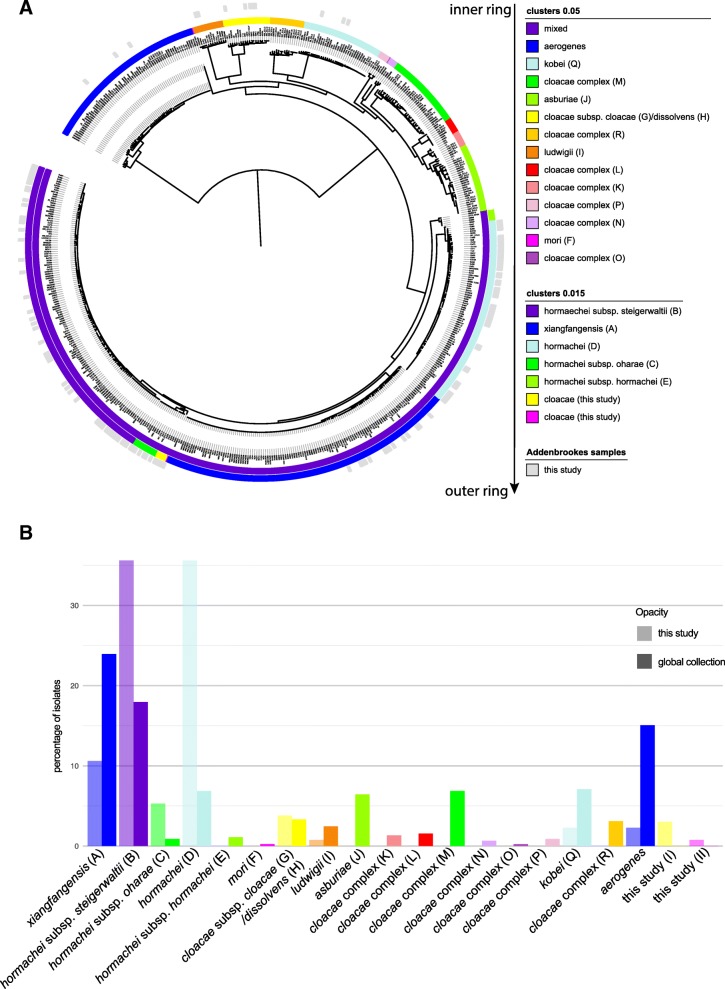
*E. cloacae* isolates from CUH within the global population structure. **a** Phylogenetic tree reconstruction from the core gene alignment highlights the diversity encountered in routine hospital samples. The inner and middle rings show the same clustering information at 0.05 and 0.015, respectively. The outer ring displays which samples are derived from this study, embedded in the global diversity [11]. Accession numbers and cluster annotation are given in Additional file 2: Tables S2 and S5 for the global collection and the samples from this study, respectively. **b** Barplot illustrating the numbers of the different lineages observed in our collection compared to the global collection [11]

*E. cloacae* contained two separate species (*E. aerogenes*, recently reclassified as *Klebsiella aerogenes* [15], and *E. kobei*), as well as several subspecies and clusters. Using the revised *E. cloacae* taxonomy [11], we observed high levels of genomic similarity. Clustering using a distance matrix (mash [13]) at the cutoff of 0.05% which is representative of the species cutoff ANI 0.95% [13] showed a large group comprised of several previously described subgroups [11] that were resolved when a finer distance cutoff (0.015) was used (excepting group G/H; Fig. 3a, Additional file 2: Table S4). The CUH population diversity was comparable to that observed for isolates from across the UK [16] (Additional file 1: Figure S3B; Additional file 2: Table S3), but unlike *K. pneumoniae*, there were clear differences evident when compared with the global collection [11] (Fig. 3b, Additional file 2: Table S5). We identified 8 out of the 18 clusters described previously [11] and discovered two new clusters nested in the widely distributed *E. xianfangensis* and *E. hormaechei* subgroups (Fig. 3a). We also observed a far lower percentage of *E. xiangfangensis* that expanded with the spread of KPC-type carbapenemases, which is consistent with our dataset lacking carbapenem-resistant isolates (Fig. 3b). Analysis of the deep branching lineages showed *E. kobei* and *E. ludwigii* were exclusively isolated from blood, whereas *E. aerogenes* and groups G/H were from a wider variety of isolation sites, possibly more indicative of opportunistic infections (Additional file 1: Figure S3C). These differences were also reflected in the predicted (genotypic) resistance profiles, with *E. kobeii* showing resistance to two or three antimicrobial classes, whereas the other deep lineages showed no (*E. aerogenes*, *E. ludwigii*) or only a low proportion (groups G/H) of MDR isolates (Additional file 1: Figure S3D).

### *K. pneumoniae* and *Enterobacter* represented flowing vs static populations in CUH between 2006 and 2012

The dominance of four globally important clones (STs: 15; 101; 307; 874) amongst the *K. pneumoniae* at CUH (41%) (Additional file 1: Figure S3A; Additional file 2: Table S1) was led by ST15 which is strongly linked to outbreaks in hospitals [8, 17, 18]. ST101 has been highlighted for its association with extensive resistance and KPC carbapenemases [19], and ST307 was reported in a recent large-scale analysis of carbapenem-resistant isolates [20, 21]. By contrast in *E. cloacae*, the main four sequence types constituted only 27% of the isolates (Additional file 1: Figure S3B; Additional file 2: Table S4), suggestive of different population dynamics for these two genera in a hospital setting.

**Fig. 4:**
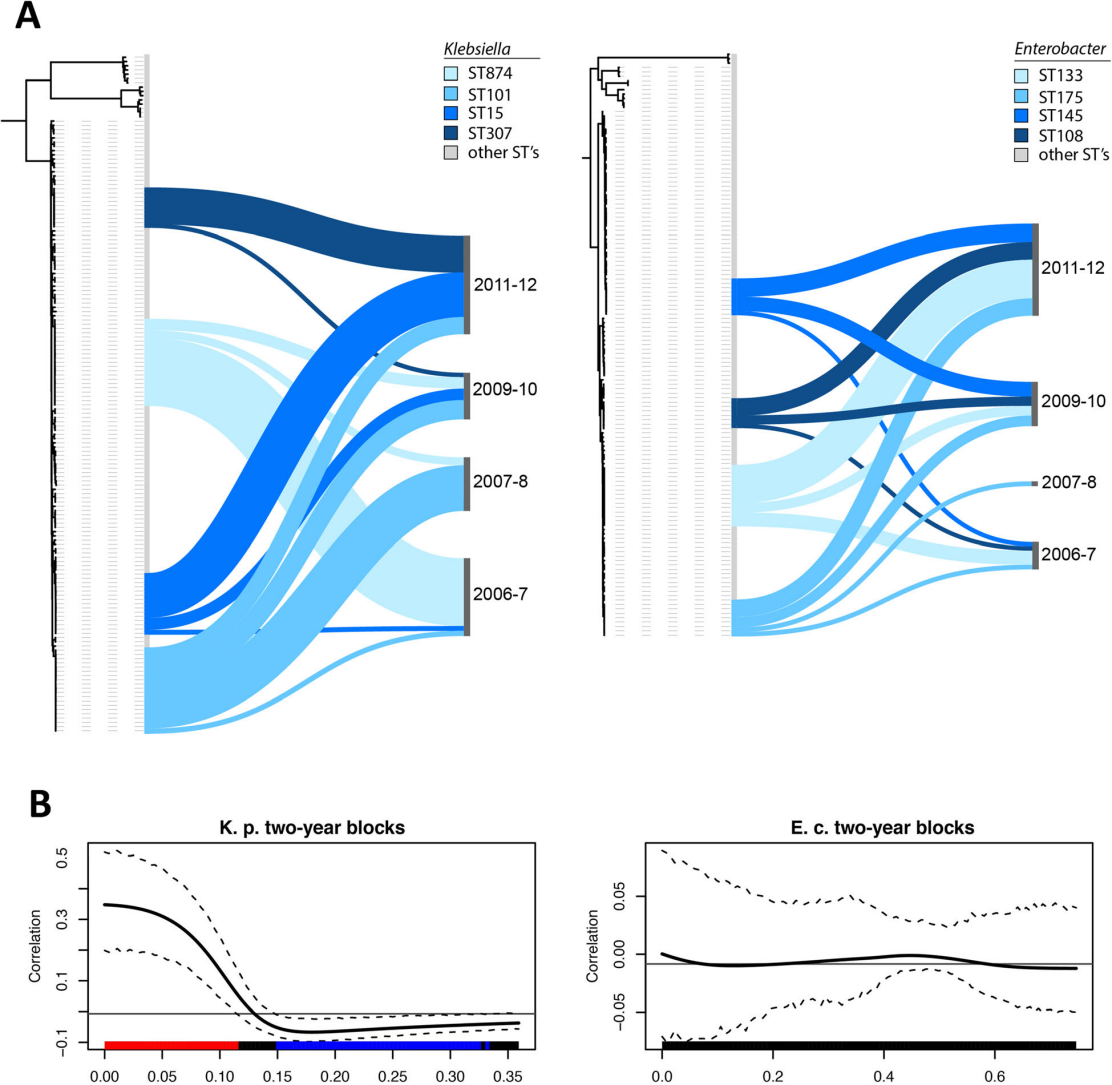
Population structures of *Klebsiella* and *Enterobacter* over time. **a** Visualizing the occurrence of the four main sequence types for Klebsiella (left tree) and *Enterobacter* (right tree) highlights a consecutive replacement of the different *Klebsiella* sequence types, whereas the main *Enterobacter* sequence types occur continuously as mixed population during all time frames. **b** Test for the correlation of the distance on the tree between two samples using SNP data from whole-genome trees after removing recombination and sharing or difference of a trait (2-year spans of isolation as given in Additional file 2: Tables S1 and S4). This shows a strong positive correlation (similar trait is more likely) for very close strains and negative correlation (similar traits are more unlikely) for large distances on the tree for *Klebsiella* (left panel); no signal can be observed for *Enterobacter* (right panel)

Successive replacements of the *K. pneumoniae* STs occurred over time (Fig. 4a). Testing for the correlation between genetic distance using whole-genome SNP data (after removing recombination) and the date of isolation showed a strong correlation between phylogenetic signal and the temporal information, using month, year, and the observed 2-year span as a trait (Fig. 4b; Additional file 1: Figure S4). This trend was not apparent for *Enterobacter* isolates (Fig. 4b), with all clones represented across the study period. These temporal signals for strain flow vs stasis between the species were not perturbed after 2007 when the change in antimicrobial prescribing policy occurred.

### Sharing of the same composite and dynamic resistance elements differed across the genera

**Fig. 5:**
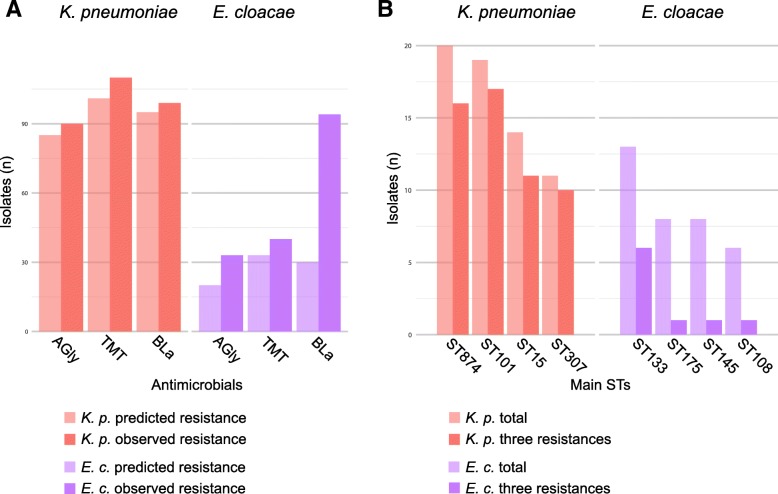
Different population and resistance profiles in the two data collections. **a** A comparison of the number of isolates to be resistant according to the genes as predicted (light bars) and measured to be resistant in physical tests (dark bars). **b** The distribution of MDR isolates across the main STs for *Klebsiella* and *Enterobacter* spp., with the total number of isolates for the ST in lighter and the number of isolates resistant against all three classes in darker color

To gain insight into the contrasting population and resistance dynamics between *K. pneumoniae* and *E. cloacae*, we compared the patterns of genotypic to phenotypic resistance, i.e., how well-predicted resistance genes could explain the observed resistance patterns (Fig. 5a).

For *K. pneumoniae*, resistance to aminoglycosides, trimethoprim, and the beta-lactams was closely linked with horizontally acquired genes (Fig. 5a; Additional file 1: Figure S5; Additional file 2: Table S1). Amongst the beta-lactams, ESBLs were detected in almost all cephalosporin-resistant isolates (95 out of 99), most often *bla*_CTX-M-15_ (85 isolates). Only three isolates encoded carbapenemases (Additional file 2: Table S1). For the same three antimicrobial classes in *E. cloacae*, gene presence predicted resistance in 20 out of 33 aminoglycoside-resistant and 33 out of 40 trimethoprim-resistant isolates (Fig. 5a, Additional file 2: Table S1 and Table S4). The poor correlation between resistance to third-generation cephalosporins and the presence of ESBL genes (30 of 94 resistant isolates acquired ESBL genes) (Additional file 2: Table S2) in *E. cloacae* could be explained by the upregulation of the endogenous AmpC beta-lactamase [22–29].

Differences are also clearly apparent when assessing the most dominant STs. Whilst *in K. pneumoniae*, the four dominant STs are almost exclusively comprised of multidrug-resistant isolates, the opposite was true for *E. cloacae* (Fig. 5b).

**Fig. 6:**
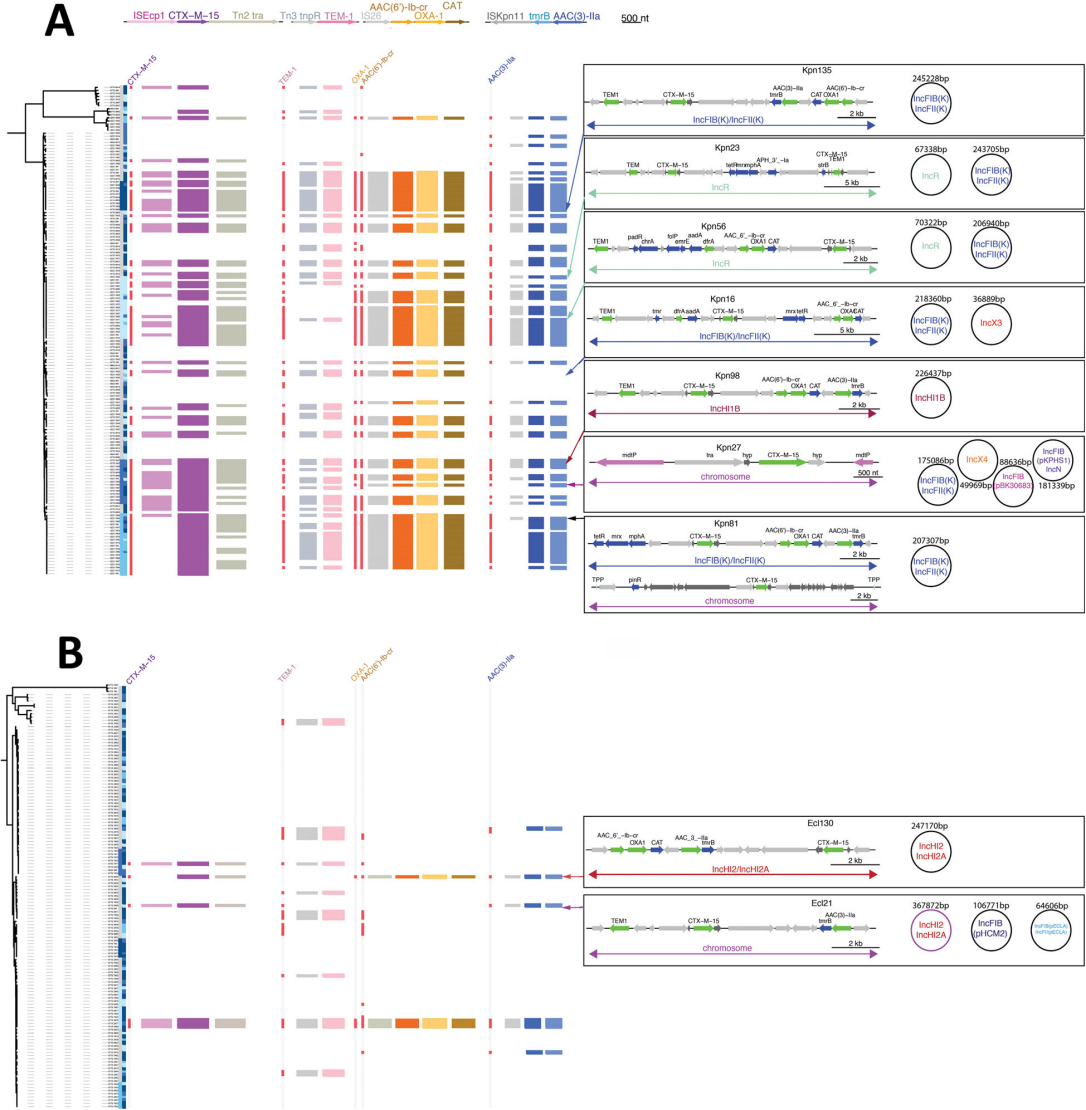
The conservation of integrons and mobile units across the species vs the dynamic broader genetic context. The guidance trees are based on the core gene alignment for *Klebsiella pneumoniae* (**a**) and *Enterobacter cloacae* (**b**). The conservation of the genetic environment of the main acquired resistance genes *bla*_CTX-M-15_, *bla*_TEM1_, *bla*_OXA1_, *aac*(6′)-*Ib*-*cr*, and *aac*(3)-*IIa* was tested by mapping against the predominantly observed cassettes surrounding these genes as mobile units. Whilst *bla*_CTX-M-15_ moves by itself in the Tn*2021* structure, and *bla*_TEM-1_ moves with a *tnpR* gene, the *bla*_OXA-1_, *aac*(6′)-*Ib*-*cr*, and *cat* (chloramphenicol-resistance) genes are conserved in an integron element and the *aac*(3)-*IIa* and *tmrB* (tunicamycin resistance) genes are conserved together. This highlights that even though there is high variability in the plasmid content, there is a core set of resistance gene cassettes that is stable in both populations, and strains very often carry all of them combined. Long-read assemblies (Pacbio) resolved the plasmid content and location of the CTX-M-15 gene in several representatives of *Enterobacter* spp. and *Klebsiella* spp., as indicated by the arrows to the core gene guide trees. The respective boxes illustrate the plasmid content with the Inc-type and their respective sizes. The arrow diagrams illustrate the location of the CTX-M-15 gene, which is highly mobile, and can be seen to be integrated into the chromosomes in three out of our nine examples, each at different locations, as well as on plasmids belonging to the IncR, IncFIB(K)/IncFII_K_, IncHI2/IncHI2A, and IncHIB types

When analyzing co-occurrence of resistance elements, we noticed that the main resistance cassettes were shared between *K. pneumoniae* and *E. cloacae* and almost always show perfect co-occurrence (Fig. 6, left panels). This includes the comparatively few *E. cloacae* encoding ESBL genes (*bla*_CTX-M-15_), which were identified with the same pattern of aminoglycoside and trimethoprim resistance genes that was found in *K. pneumoniae*, indicating both species access the same shared gene pool, but the distribution across the populations is clearly different between the two species*.* To resolve this, we performed long-read (PacBio) sequencing on selected isolates, which confirmed that the resistance genes shared related genetic environments, suggesting that both genera were acquiring a similar pool of resistance genes. However, there is an extensive pool of mobile elements being sampled, with no two isolates sharing the same plasmid repertoire (Fig. 6 right panels). The key ESBL gene *bla*_CTX-M-15_ was found integrated at an array of different chromosomal sites or carried on a diverse set of plasmid replicons in *K. pneumoniae* and *E. cloacae* alike (Fig. 6a, b), with no two strains sharing it at the same location. A comparison against a curated large-scale plasmid database showed that these elements co-occur (Additional file 1: Figure S6), but less frequently as seen in our examples. This is most likely since similar geographic and temporal isolation sources seem to reflect a dynamic pool of transposable elements and plasmids shared between *K. pneumoniae* and *E. cloacae*, and potentially also other members of the *Enterobacterales* occurring at the same time in the area.

## Discussion

WGS highlighted the contrasting AMR populations of these species in our hospital across 6 years from 2006. The *K. pneumoniae* population was dominated by clonal replacements of major STs with the majority of isolates multidrug-resistant due to uptake of resistance plasmids (STs 874, 101 and 307), whilst the *E. cloacae* hospital population reflected a continuous sampling from the whole diversity without temporal signal and without apparent fitness advantage for strains carrying mobile elements conferring resistance. For *Klebsiella*, this indicated the importance of horizontal acquisition of ESBLs, like *bla*_CTX-M-15_, whilst for *Enterobacter*, it emphasized the capacity for intrinsic cephalosporin resistance via the endogenous AmpC enzyme [30].

We observed four successive introductions of highly cephalosporin-resistant *K. pneumoniae* clones. Three of the four (excepting ST307) are known to be widespread in the UK [14], and all four have been seen to be resistant lineages disseminated internationally [20]. In contrast, for *Enterobacter*, there were differences between this cephalosporin resistance-focussed study and a recent global study. The main difference was due to an under-representation *E. xiangfangensis,* a major *Enterobacter* lineage associated with plasmid-acquired KPC-based carbapenem resistance. This is in accordance with our sampling time frame, which are derived from times before carbapenems were widely used, and strongly indicates that the shift from intrinsic resistance to advanced cephalosporins (in our study) to relying on acquired resistance mechanisms for carbapenem resistance (via plasmid-derived KPC) changed the *E. cloacae* population structure towards few dominating clones, as discussed in [16].

The finding that small numbers of diverse *Enterobacter* acquired the same collection of mobile resistance elements found across *K. pneumoniae* demonstrated a similarity between the species in their use of a linked network of mobile resistance genes, which, based on reports on *E. coli* [3], is widely shared with other *Enterobacterales*. For this study, the consistent profile of the numerous resistance genes present alongside *bla*_CTX-M-15_ detected from short-read data masked the extensive plasticity and variability of the complex resistance elements acquired by both species. Despite this plasticity of different plasmids and chromosomal integration sites, the presence and absence of the resistance genes themselves remained stable across the major *K. pneumoniae* clones found in 2012 (ST307) or in 2006 (i.e., ST874). The lack of association between the persistent or successful lineages of *E. cloacae* indicated that AMR alone did not explain the success of major clones in either genus, a point that will form the basis of ongoing and future work.

## Conclusions

We demonstrated the stark contrast of the MDR population dynamics between two major pathogens in a major UK hospital. Whilst cephalosporin resistance in *K. pneumoniae* is plasmid driven, *E. cloacae* can rely on its chromosomal resistance mechanism. This leads to a markedly different population structure and shows that plasmid-derived resistance leads to the shift from a highly diverse population to the dominance of a few major lineages, whilst chromosomal resistance mechanisms do not bias the population, with a similar composition observed throughout the 7-year time frame.

## Materials and methods

### Surveillance data for national context

The annual national context for cephalosporin and aminoglycoside resistance in *K. pneumoniae* and *E. cloacae* was analyzed using antimicrobial susceptibility data from the BSAC Bacteraemia Resistance Surveillance Programme (www.bsacsurv.org, accessed April 2018). Susceptibility data for gentamicin, cefotaxime, and ceftazidime from the first seven isolates of both species collected at each of 30 sentinel laboratories each year between 2001 and 2012 was included.

### Clinical setting, patient data, and isolate collections

Cambridge University Hospitals NHS Foundation Trust (CUH) is a 1000-bed secondary and tertiary referral hospital. The on-site Public Health England Clinical Microbiology Laboratory processes ca. 1,000,000 specimens per annum for CUH. Linked anonymized patient demographic and routine microbiology laboratory data (NHS research ethics committee approval 12/SC/0431), including species identifications by API (bioMérieux, Marcy L’Etoile, France) or MALDI Biotyper (Bruker Daltonics, Bremen, GmbH) indicated that between 2006 and 2012, 13,379 patients were positive for *K. pneumoniae* (from any specimen type) and 5661 patients were positive for *E. cloacae* (from any specimen type). We included and analyzed antimicrobial susceptibility data, determined according to the BSAC disc diffusion method [31] for the first isolate of these two species from each patient.

Between 2006 and 2012, *K. pneumoniae* and/or *E. cloacae* associated with invasive disease (isolated via sterile site cultures) in patients at CUH was stored at − 80 °C. To understand the populations of sensitive and resistant isolates of each species by WGS, we first stratified the 465 invasive *K. pneumoniae* and 225 invasive *E. cloacae* according to their routine laboratory antimicrobial susceptibility results for six groups of antimicrobials that were tested each year between 2006 and 2012 (penicillins, amoxicillin-clavulanate, aminoglycosides, fluoroquinolones, trimethoprim, and third-generation cephalosporins). To enrich for drug-resistant isolates, we included all of the available invasive isolates that were resistant to three or more groups for both species (Table 1). From amongst the invasive isolates that were resistant to two groups of antimicrobials or fewer, we randomly selected 44 *K. pneumoniae* isolates (11.5% of the available isolates) and 28 (18.4%) of the available *E. cloacae* isolates (Table 1), which for the invasive isolates, represented 20–34% of *K. pneumoniae* and 40–45% of *E. cloacae* isolated in any year being included for study. Where a patient had an isolate of both species that was resistant to three or more groups of antimicrobial, we included one isolate of both species. Isolates of the same species were de-duplicated to represent one isolate per species per patient except four patients for whom two isolates of *K. pneumoniae* were included due to differences in initial phenotypic susceptibilities that were considered suggestive of different strain types. From this collection of invasive isolates, we included 124 *K. pneumoniae* from 120 patients and 94 *E. cloacae* from 94 patients for further study. Additionally, a prospective collection (during the last half of 2012) of *K. pneumoniae* and *E. cloacae* isolated from samples taken from any body site yielded a further 38 isolates of *K. pneumoniae* and 35 isolates of *E. cloacae* from a total of 73 patients. Of these, 33 *K. pneumoniae* and 26 *E. cloacae* isolates were resistant to three or more groups of antimicrobials (Table 1).

Of the isolates included for study, four carbapenemase-producing *E. cloacae* were identified by Public Health England’s (PHE) national Antimicrobial Resistance and Healthcare Associated Infections (AMRHAI) Reference Unit to produce IMP-1 carbapenemase (as well as other co-acquired resistance genes). One *K. pneumoniae* was identified later to carry the NDM-1 carbapenemase gene, one to carry the KPC-2 gene, and one the CARB-12 gene. In total, 162 *K. pneumoniae* from 158 patients and 133 *E. cloacae* from 133 patients were sequenced (Additional file 2: Table S1 and S4).

### Bacterial culture and antimicrobial susceptibility testing

Clinical specimens had been previously processed according to standard operating procedures, including antimicrobial susceptibility testing using the BSAC disc diffusion method [31] with results interpreted using EUCAST breakpoints. For this study, bacteria were re-identified using MALDI-ToF (Bruker Daltonics) and antimicrobial susceptibilities confirmed with the VITEK-2 instrument (BioMérieux). MICs were determined by agar dilution methodology for three NDM carbapenemase-producing *E. cloacae* in Public Health England’s AntiMicrobial Resistance and Healthcare Associated Infections (AMRHAI) Reference Unit.

### DNA sequencing

Isolates were cultured overnight on Brilliance UTI chromogenic agar (Oxoid, UK), then inoculated into Brain Heart Infusion broth (Oxoid, UK) and grown to stationary phase. DNA was extracted and quantified [8]. Whole-genome sequencing was performed on a HiSeq 2000, and 150 bp paired-end Illumina sequencing reads were produced [32]. Selected samples were also sequenced on a PacBio RSII using P6/C4 sequencing chemistry. High molecular weight DNA for long-read sequencing was prepared from bacterial cultures by phenol-chloroform extraction from overnight cultures; phases were separated by centrifugation using MaXtract phase lock tubes (Qiagen) according to the manufacturer’s instructions. Libraries were made using the SMRTbell Template Prep Kit 1.0. Sequence data have been deposited in the European Nucleotide Archive (Additional file 2: Table S1 and S2).

Annotated assemblies of short-read Illumina data were produced [33]: briefly, for each sample, multiple assemblies were created using VelvetOptimiser v2.2.5 [34] and Velvet v1.2 [35], assembly improvement using the best N50 and contigs in scaffolds via SSPACE [36] was followed by sequence gap filling using GapFiller [37]. For PacBio, sequence reads were assembled using Canu (v1.1) [38] with filtered subreads generated through the SMRT analysis software v2.3.0. Assemblies were circularized using Circlator v1.1.3 [39] using the corrected reads generated during the Canu assembly. Finally, the circularized assembly was polished with the short-reads and pilon as implemented in the unicycler-polish script [40]. If not all resulting contigs were circularized, assemblies combining the Illumina and PacBio reads were performed using unicycler [40], followed by Circulator with the corrected reads generated in the previous Canu assembly, and unicycler-polish. The assemblies used were based on Canu for Kpn135, Kpn23, Kpn27, Kpn56, Kpn81, Kpn98, Ecl21, and Ecl55; the hybrid assembly was used for Kpn16 and Ecl130. All assemblies were controlled by mapping back the PacBio and Illumina reads of the respective strain using bwa mem (default options; -x pacbio for the pacbio reads), as well as Mauve alignments of the Illumina assemblies to the final assemblies. Automated annotation of all assemblies was performed using PROKKA v1.11 [41] and genus-specific databases from RefSeq [42].

### Sequence analyses

Multi-locus sequence typing was performed using blast against the sequence types available at PubMLST [43]. The resistance gene content and plasmid replicon types carried by each isolate were predicted using the short-read search software ariba [44] with the implemented arg-annot as provided for SRST2 [45, 46] and PlasmidFinder [47] databases respectively. *K. pneumoniae* capsule and O-antigen type was predicted using Kaptive [48], using the capsule type database provided with Kaptive and an in-house generated database based on the different O-antigen operons described [49] (Additional file 2: Table S2); the distinction between different O-types was revised according to the recently published updated typing online platform (http://kaptive.holtlab.net) [50].

We combined our *K. pneumoniae* and *E. cloacae* datasets with a global collection and a UK-focused collection (Additional file 2: Table S3), for each species [7, 11, 14, 16]. Roary [51] was used to determine the core, soft core, and shell components of the pan-genome for the respective datasets (Additional file 2: Tables S1 - S5). For core gene tree-based phylogeny, the core gene alignment from roary was retrieved and informative sites were chosen using snp_sites [52] and trimal [53] as detailed in the respective figure legends, and RAxML (v 8.2.8) was used to construct phylogenetic trees using the general time-reversible (GTR) model with 100 bootstrap support calculations. iTOL was used for tree and metadata visualization [54]. Clustering was performed using mash (as an approximation of ANI [13]), and cutoffs were chosen as indicated in the figure legend (Fig. 3). Reference sequences for the mash clustering to define (sub)species and further groups were used as given in Additional file 2: Table S5. (Pro)Phages in the PacBio assemblies were predicted using Phaster [55].

### Bacteria mapping and variant detection

For each sample of *K. pneumoniae* or *E. cloacae*, sequence reads were mapped against the reference genome (*Enterobacter hormaechei* subsp. *steigerwaltii* DSM 16691, Genbank accession CP017179.1, for *Enterobacter*; *K. pneumoniae* subsp. *pneumoniae* MGH 78578, Genbank accession CP000647.1, for *Klebsiella*) using SMALT v0.7.4 [56, 57] to produce a BAM file. Variation detection was performed using SAMtools mpileup v0.1.19 [58] with parameters “-d 1000 -DSugBf” and bcftools v0.1.19 [59] to produce a BCF file of all variant sites. The option to call genotypes at variant sites was passed to the bcftools call. All bases were filtered to remove those with uncertainty in the base call. The bcftools variant quality score was required to be greater than 50 (quality < 50) and mapping quality greater than 30 (map_quality < 30). If not all reads gave the same base call, the allele frequency, as calculated by bcftools, was required to be either 0 for bases called the same as the reference, or 1 for bases called as a SNP (af1 < 0.95). The majority base call was required to be present in at least 75% of reads mapping at the base (ratio < 0.75), and the minimum mapping depth required was 4 reads, at least two of which had to map to each strand (depth < 4, depth_strand < 2). Finally, strand_bias was required to be less than 0.001, map_bias less than 0.001, and tail_bias less than 0.001. If any of these filters were not met, the base was called as uncertain.

A pseudo-genome was constructed by substituting the base call at each site (variant and non-variant) in the BCF file into the reference genome, and any site called as uncertain was substituted with an N. Insertions with respect to the reference genome were ignored, and deletions with respect to the reference genome were filled with N’s in the pseudo-genome to keep it aligned and the same length as the reference genome used for read mapping. Gubbins [57] was then used to remove recombinant/highly variable regions from the alignment, and the resulting reduced alignment was used as input for tree calculations as well as pairwise SNP comparisons. These were performed with the dist.alignment() function in the R package seqinr [60], which gives relative values and therefore enables plotting both organisms onto one *y*-axis. We tested for relative enrichment of organisms of the same sequence type vs. different sequence type in the same or different 2-year time span using the chi.square() function as implemented in R (v3.4.2). The contingency matrix consisted of numbers of isolates of same vs. different ST occurring in the same vs. different 2-year span, which was significant for *K. pneumoniae* and not significant for *E. cloacae* (*p* values < 2.2e−16 and *p* = 0.03, respectively).

The masked alignment was used as input for phylogenetic tree calculation using RAxML as described above, disregarding positions with more than 5% N content, to perform tests for phylogenetic signal, where 10 maximum likelihood trees were generated as described above. For branch length comparisons and testing for phylogenetic signal, we used the phylosignal package in R and calculated correlation plots using either month, year, or 2-year span as trait information [61]. To ensure that enough signal is in these three traits, for each correlation, we also performed a randomization step using the sample() function in R. All tree files and alignments as well as the R script used to assess phylogenetic signal, SNP distances, and the chi-square test are available for download at the figshare link [https://figshare.com/s/830209f84c587c28813e].

#### Plasmid/integron analyses

Pacbio plasmids were annotated using the online plasmidfinder database to annotate the assembled sequences. The integron/mobile cassette sequence(s) were identified manually by searching for the main beta-lactam and aminoglycoside resistance conferring genes (*bla*_OXA_, *bla*_CTX_, *bla*_TEM_, *aac*(3)IIa, *aac*(6′)-Ib-cr), and annotation was performed by using the ISFinder resource [62] as well as blast against the non-redundant genbank database [63, 64]. Annotating the identified mobile units in the Illumina samples was performed by blasting the DNA sequences derived from pacbio against the Illumina assemblies with an *e* value cutoff of 1e−90; partial hits were identified as encoding the respective resistance genes, but truncated mobile elements due to frameshifts or other elements inserting within them. Comparison of the operons was depicted using GenePlotR [65] with the respective closest reference contigs as templates; replicons were identified using the plasmidfinder resource as described above. For a comparison against the plasmid database, the assemblies of the database were downloaded (16. 04. 2019; 10.15146/R33X2J) [66], and blast runs performed using the same elements as in the ariba search as input. To account for less closely related organisms, we accepted hits with over 99% similarity. If several hits of the same cassette on one plasmid were reported, the longest element was chosen; if two elements were still of the same length, the element with the higher similarity was chosen; if this still did not distinguish the hits, the first one in the sequence was chosen for representation. The plasmids were clustered using the heatmap.2() command in R using the percentage of similarity to the different cassettes as data matrix for clustering. The region of coverage on the cassettes was displayed using the ggtree package and the facetplot function in R [67].

## Additional files

Additional file 1: **Figure S1.** Comparison of sources of isolation for *K. pneumoniae* and *E. cloacae* datasets. **Figure S2.** Addenbrookes hospital isolates reflect the UK-wide diversity of isolates. **Figure S3.** *K. quasipneumoniae* and *K. variicola* are mostly part of the sensitive population of *Klebsiella,* amongst *Enterobacter E. aerogenes*, *E. kobei*, *E. ludwigii* and *E. dissolvans* show varying patterns of isolation site and drug resistance. **Figure S4.** Testing for phylogenetic signal. **Figure S5.** Number of resistance genes in sensitive and resistant populations. Figure S6. Comparison of study isolates against a curated large-scale plasmid database. (DOCX 1489 kb)

Additional file 2: **Table S1.** Accession numbers, isolation site, study month of isolation, detected acquired resistance genes, antimicrobial resistance phenotypes, of *Klebsiella pneumoniae* in this study. **Table S2.** Accession numbers and metadata for the global *K. pneumoniae* collection [7]*.* **Table S3.** Accession numbers and metadata for UK *K. pneumoniae* and *E. cloacae* [14, 16]*.* **Table S4.** Accession numbers, isolation site, study month of isolation, detected acquired resistance genes, antimicrobial resistance phenotypes, of *Enterobacter cloacae* in this study. **Table S5.** Accession numbers and metadata for the global *E. cloacae* collection [11]. (XLSX 176 kb)

Additional file 3: Review history. (DOCX 15 kb)

## Data Availability

All data generated or analyzed during this study are included in this published article and its additional files. The sequence data generated in this study are publicly available in the NCBI Archive under the accession code PRJEB1271 [68]. Accession numbers for all genomes sequenced in this study are available in Additional file 2 S1 (*K. pneumoniae*) and S4 (*Enterobacter*). The accessions are given alongside key metadata including clinical isolation site, year of isolation, bacterial MLST assignations, and resistance gene detections in the Additional file 2 and FIGSHARE dataset [69].
