# Supplementary material for: Contrasting patterns of longitudinal population dynamics and antimicrobial resistance mechanisms in two priority bacterial pathogens over 7 years in a single center
Source: Genome Biol. 2019 Sep 2;20:184. doi: 10.1186/s13059-019-1785-1 (PMC6717969; doi:10.1186/s13059-019-1785-1)
Supplement: Supplementary file 1 — Figure S1. Comparison of sources of isolation for K. pneumoniae and E. cloacae datasets. Figure S2. Addenbrookes hospital isolates reflect the UK-wide diversity of isolates. Figure S3. K. quasipneumoniae and K. variicola are mostly part of the sensitive population of Klebsiella, amongst Enterobacter E. aerogenes, E. kobei, E. ludwigii and E. dissolvans show varying patterns of isolation site and drug resistance. Figure S4. Testing for phylogenetic signal. Figure S5. Number of resistance genes in sensitive and resistant populations. Figure S6. Comparison of study isolates against a curated large-scale plasmid database. (DOCX 1489 kb) [file 13059_2019_1785_MOESM1_ESM.docx]

**
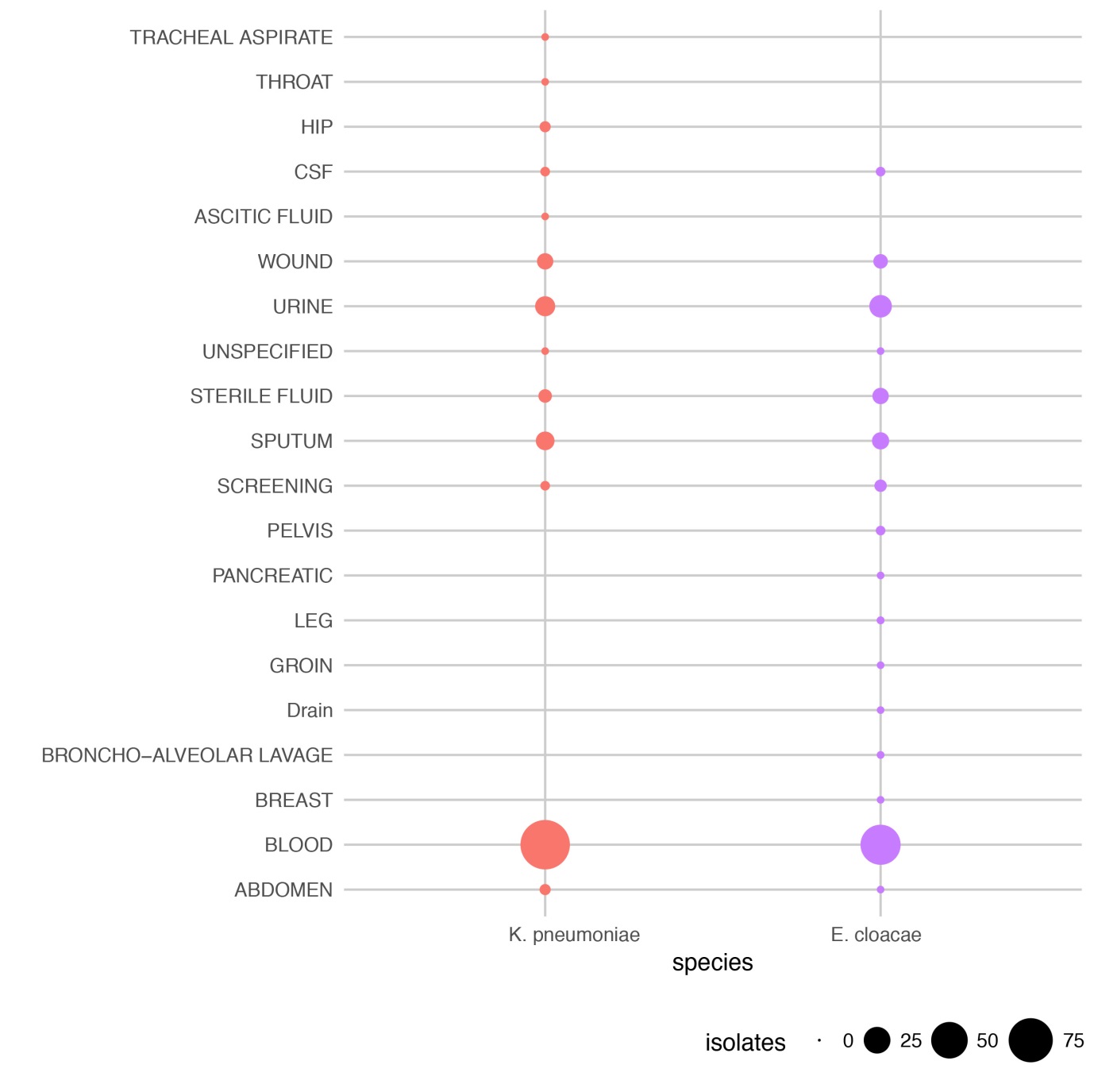
**

**Fig. S1. Comparison of sources of isolation for *K. pneumoniae* and *E. cloacae* datasets.**

**
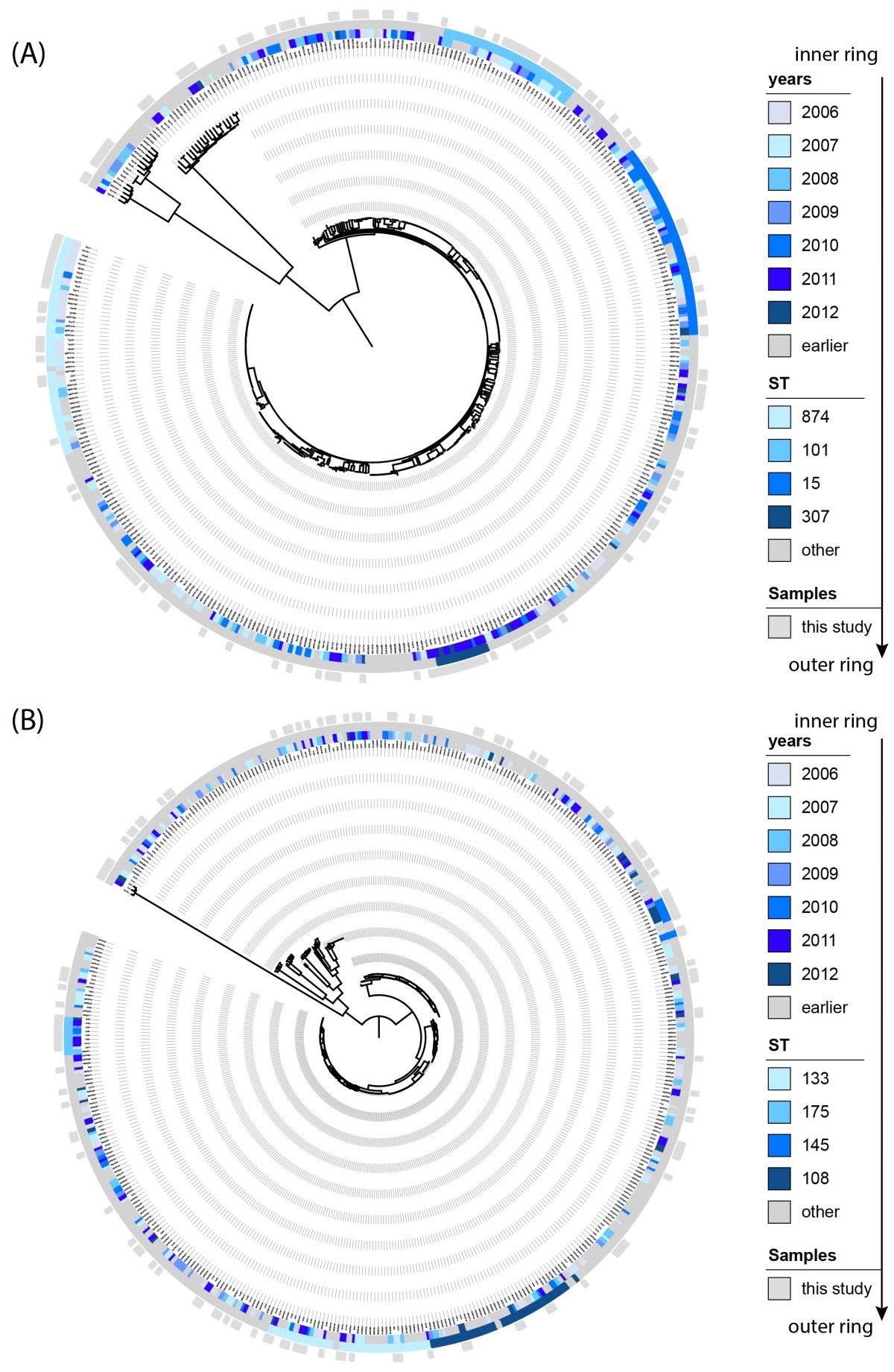
**

**Fig. S2. Addenbrookes hospital isolates reflect the UK-wide diversity of isolates.** Phylogenetic tree combining a UK-wide collection focused on multidrug-resistant isolates between 2000-2011 (*1, 2*) and our data, demonstrating that both our set of *Klebsiella* *pneumoniae* (A) and *Enterobacter cloacae* (B) are representative of the wider UK populations [14, 16].


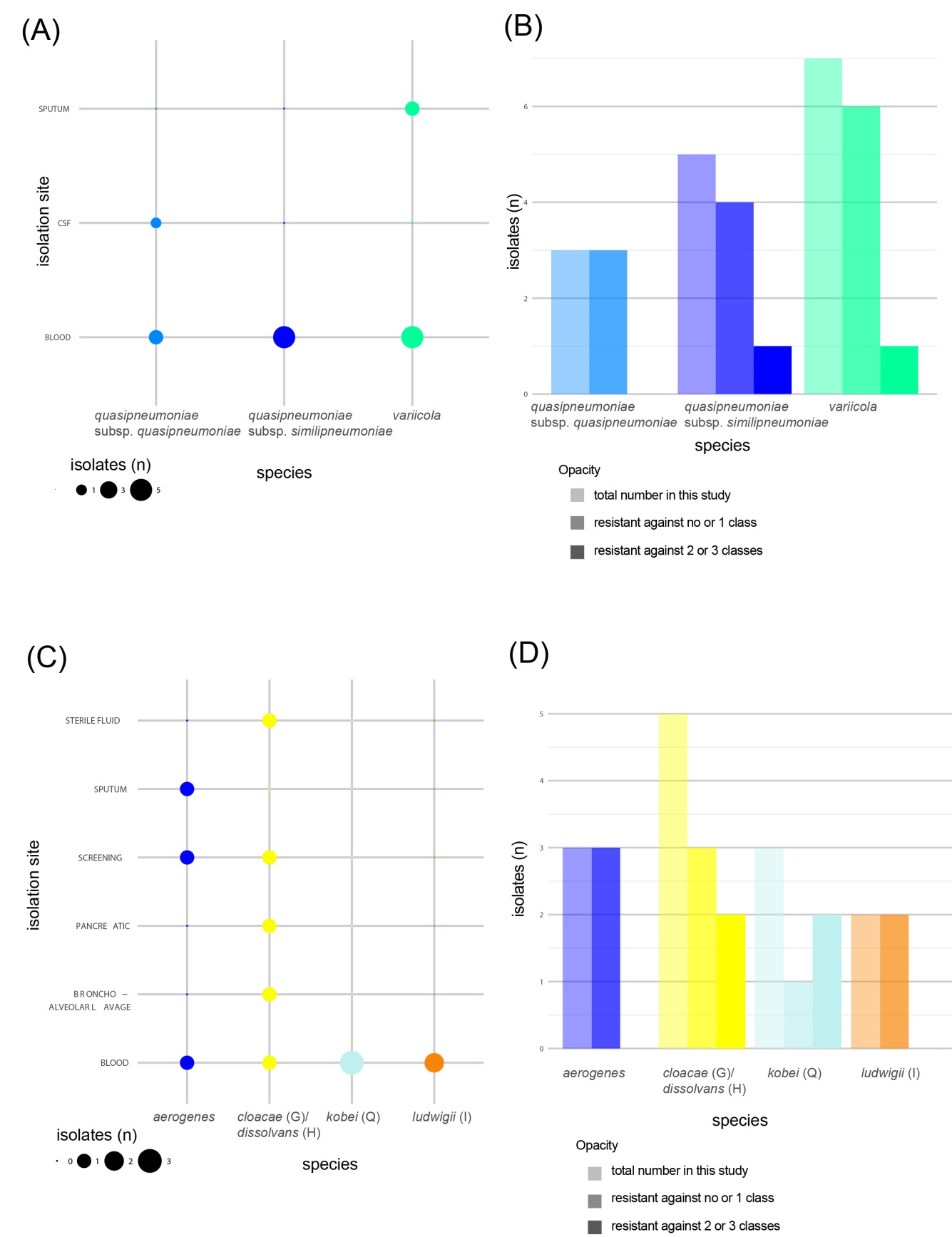


**Fig. S3. *K. quasipneumoniae* and *K. variicola* are mostly part of the sensitive population of *Klebsiella,* among *Enterobacter* *E. aerogenes*, *E. kobei*, *E. ludwigii* and *E. dissolvans* show varying patterns of isolation site and drug resistance.** Detailed analysis of the less prevalent species of the *K. pneumoniae* complex shows their site of isolation (A) and drug resistance profiles based on phenotypic susceptibility test results (B). Whilst the detailed analysis of the less prevalent species of the *E. cloacae* complex shows their site of isolation (C) and drug resistance profiles based on phenotypic susceptibility test results (D).


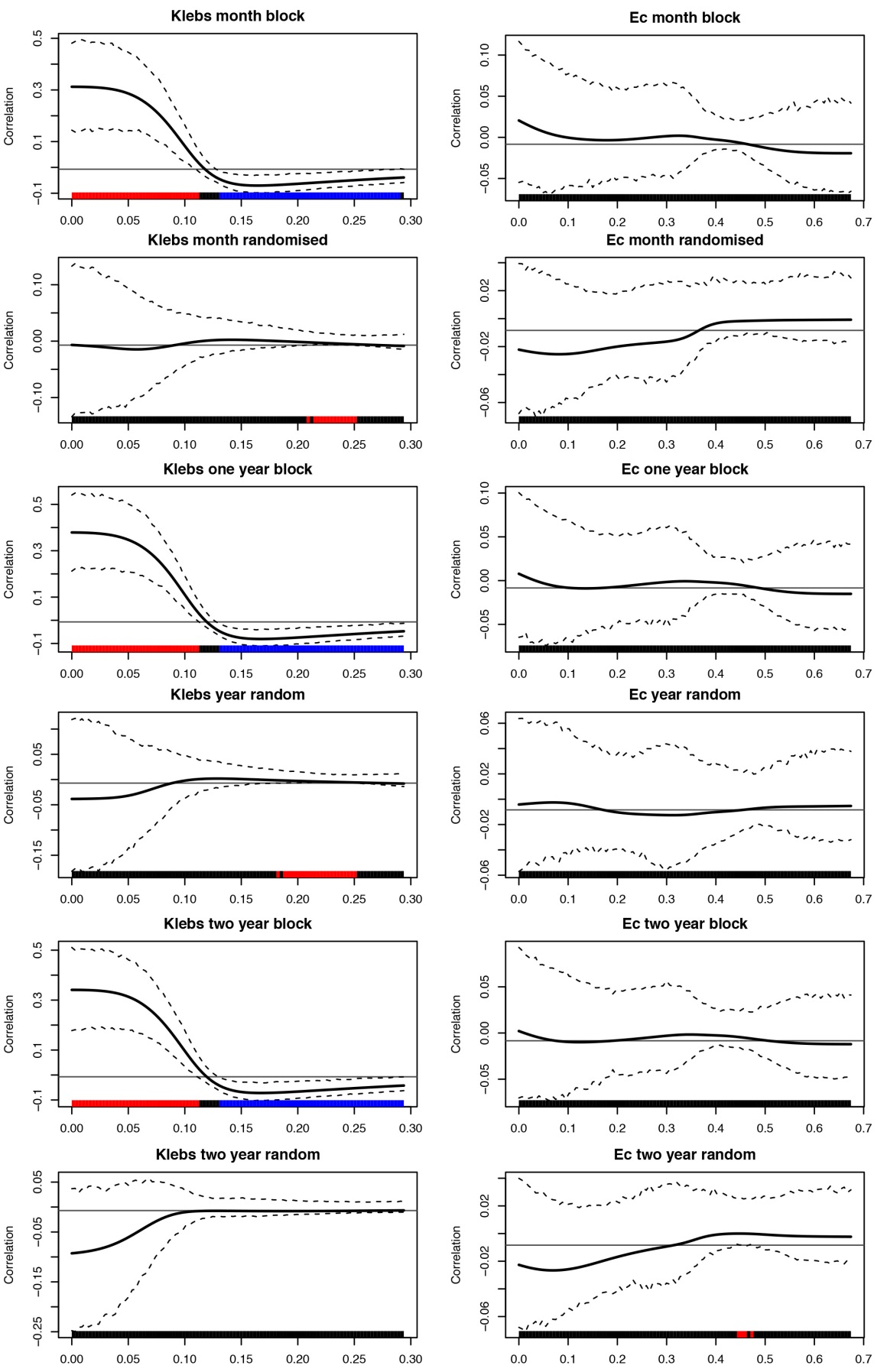


**Fig. S4. Testing for phylogenetic signal.** Data is shown for trees from *Klebsiella pneumoniae* (left column) and *Enterobacter cloacae* (right column), testing for phylogenetic signal as described above using the months, years and two-year spans as described in the methods. For each test, a second plot is shown where the trait (month, year or two-year span as indicated in the main legend) was randomized, which shows that the correlation signals observed for *Klebsiella* with all three different traits is not an artefact of the tree structure or insufficient signal in the trait, as no correlation can be observed with the randomized data. No signal can be observed for *Enterobacter* for either the randomized or the original trait data, again highlighting the different population dynamics of these two organisms.


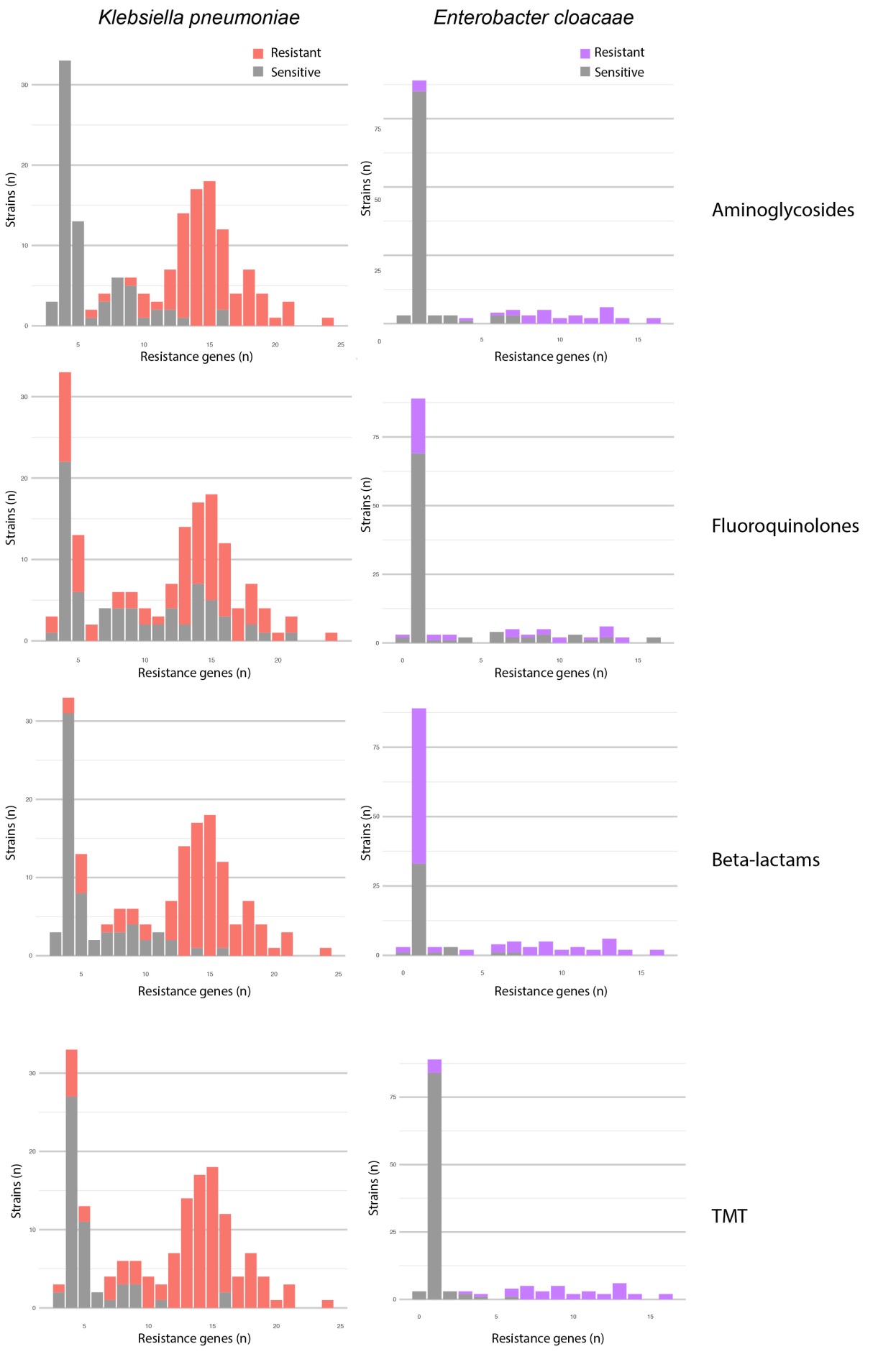


**Fig. S5. Number of resistance genes in sensitive and resistant populations.** Comparison of the numbers (y-axis) of resistant (red/violet) and sensitive (grey) strains for *K. pneumoniae* (red, left panel) and *E. cloacae* (violet, right panels) for the indicated resistances according to the number of predicted resistance genes (x-axis).

**Fig. S6.** **Variability within the resistance cassettes in *K. pneumoniae***. Revealed by comparison of study isolates against a curated large-scale plasmid database [66]. The elements can be found in the same conformation, with considerable differences in the number of bases (x axes) present for sections of DNA associated with the genes for TEM-1 (pink) CTX-M-15 (purple) *tmrB* (blue) and OXA-1 (yellow) genes.
