# Supplementary material for: Contrasting patterns of longitudinal population dynamics and antimicrobial resistance mechanisms in two priority bacterial pathogens over 7 years in a single center
Source: Genome Biol. 2019 Sep 2;20:184. doi: 10.1186/s13059-019-1785-1 (PMC6717969; doi:10.1186/s13059-019-1785-1)
Supplement: Supplementary file 3 — Review history. (DOCX 15 kb) [file 13059_2019_1785_MOESM3_ESM.docx]

Review History

**First round of review**

**Reviewer 1**

**Are you able to assess all statistics in the manuscript, including the appropriateness of statistical tests used?**

There are no statistics in the manuscript.

**Comments to author:**

This manuscript described the population dynamics of klebsiella and enterobacter spp over a 7-year period from a single UK institution. Overall, the paper is well written and the methodology is sound (for most part).

There are some major issues that need to be addressed. Please keep in mind that I am not being critical, this is a very ambitious study and I applaud the authors for their effort.

1. Due to the large amount of data, the manuscript is rather vague. I would suggest that the authors separate the klebsiella from the enterobacter into 2 papers and describe the results in more detail. There is so much than can be added to make this interesting; E.G. among the dominant clones identified in this study, were there different clades and what was the geographical distribution within the medical institution over time?

2. My biggest concern is the number of isolates that underwent WGS (Table 1 on page 13) and how they were selected to represent the total population. To determine the population dynamics of a specie over time, all the isolates from that specie needs to be included, not just over 30%. Also, there is large numbers from the Q4 2012 when compared to the rest of 2006-12 which will surely bias the results towards the later period of the study. And the selection process used in Table 1 bias the sequenced population towards being AMR (e.g. 28/29 klebsiella R to 4 antibiotics, 43/43 R to 6 antibiotics were sequenced but only 28/279 klebsiella R to 1 antibiotic were sequenced). Surely this collection does not really represent the true population dynamics of klebsiella spp at that institution.

3. The authors used mostly short-read sequencing to characterize MGEs associated with AMR. Short-read sequencing is not ideal to characterize MGEs (although I completely understand the reasoning because of cost-limitations using long-read sequencing).

Some minor comments:

1. There are confusion in the literature regarding the terms, clones, clades, clusters and lineages. The authors use lineage liberally but refer to different entities E.G. Line 83 lineage refers to different klebsiella spp and on line 42 it seems to refer to clones. Please be consistent.

2. Lines 74-80; why were the fluoroquinolones not mentioned. For enterobacter spp (less likely for klebsiella spp), this group of agents are considered as one of the 1st line treatment options for serious infections.

3. Line 159; E. aerogenes has recently been re-classified as Klebsiella aerogenes.

**Reviewer 2**

**Are you able to assess all statistics in the manuscript, including the appropriateness of statistical tests used?**

Yes, and I have assessed the statistics in my report.

**Comments to author:**

The authors present the results of a temporal analysis of MDR Klebsiella and Enterobacter from a single hospital site. The uniqueness of this study is that it allows a fine scale analysis in the fluctuations and population dynamics of these bacteria which are missed from the usual global collection studies, or even national studies which examine snapshots in time. From this they identify clear differences in population dynamics between the two species, suggesting Klebsiella fluctuates based on the cycling of globally disseminated clones carrying MDR plasmids, whilst Enterobacter is a stable population not affected by plasmid carriage and resulting clinically relevant resistance. This is largely driven by a chromosomal intrinsic ampC.

The data are well interpreted and presented, and I have a few comments below that the authors may wish to consider to provide more clarity to the paper

Major Points:

Line 137: Not really a major point, but there is a dramatic sub-selection of isolates for sequencing without any real description of why or how they were chosen. Cab we be assured they are representative of the population, especially since this is crucial in affirming that Klebsiella was more prone to circulating clones. On a similar line its not clear from the results text how in silico resistance prediction shows sensu stricto comprises the majority, especially given the clear mobility of resistance genes shown later.

Line 151: It would be nice to see major lineages (STs) designated on the Kleb tree

May main point revolves around the ESBL gene context. The section is excellently presented and makes the paper in my opinion. However i think some confirmation needs to be done. Is the observation of shared contexts because these islands co-locate on all instances of, for example, CTX-M-15? This could be checked against reference sequences on NCBI and also other long read collections such as the recent ST131 E. coli reports from some of this group. The suggestion on line 253 of shared networks of MGE is contingent upon these genes such as CTX-M-15 having the possibility of being able to be found in other contexts and needs to be confirmed. If CTX-M-15 can only be found in that single context it says nothing about sharing networks really, and only that CTX-M-15 always sits in this genetic context regardless of where it is found.

Minor points:

Line 38: should read "success of specific lineages"

Line 45: should read "led to markedly"

Line 74: suggest rewording to "both K. pneumoniae and E. cloacae remain largely susceptible to other important groups of antimicrobials in the UK"

Line 92: remove "however"

Line 94-8: Sentence is far too long and has a poor grammatical flow. Same with line 104-8.

Line 99: Can you actually measure how antimicrobial resistance as competitive advantage in a hospital setting correlates with the population structure? Not sure it is done in this paper even if you can

Line 111: Inclusion of "first" is confusing and doesnt make sense in this context. The 2.4 times more likely statement is questionable in the absence of detail of how the strains were isolated. I know this is in the methods but a succinct summary would benefit here. It needs to be clear if these are all clinical symptomatic isolates.

Line 375: please, just provide the method for assigning MLST here, paper hunts are not acceptable

**Authors Response**

**Point-by-point responses to the reviewers’ comments:**

Reviewer #1:

This manuscript described the population dynamics of klebsiella and enterobacter spp over a 7-year period from a single UK institution. Overall, the paper is well written and the methodology is sound (for most part).

There are some major issues that need to be addressed. Please keep in mind that I am not being critical, this is a very ambitious study and I applaud the authors for their effort.

1.	Due to the large amount of data, the manuscript is rather vague. I would suggest that the authors separate the klebsiella from the enterobacter into 2 papers and describe the results in more detail. There is so much than can be added to make this interesting; E.G. among the dominant clones identified in this study, were there different clades and what was the geographical distribution within the medical institution over time?

*Thank you for your comments, we respectfully disagree and feel that the unique aspect if this study was to move away from studying single pathogens longitudinally and in isolation. Hence our desire to maintain the content and format of the manuscript. We also note the additional comment made by the editor in this regard.*

2. My biggest concern is the number of isolates that underwent WGS (Table 1 on page 13) and how they were selected to represent the total population. To determine the population dynamics of a specie over time, all the isolates from that specie needs to be included, not just over 30%. Also, there is large numbers from the Q4 2012 when compared to the rest of 2006-12 which will surely bias the results towards the later period of the study. And the selection process used in Table 1 bias the sequenced population towards being AMR (e.g. 28/29 klebsiella R to 4 antibiotics, 43/43 R to 6 antibiotics were sequenced but only 28/279 klebsiella R to 1 antibiotic were sequenced). Surely this collection does not really represent the true population dynamics of klebsiella spp at that institution.

*We have emphasised that we sought to compare the flux of AMR in populations of Klebsiella and Enterobacter in the Abstract (line 47-8) and at the end of the introduction (lines 109-116). Also, we have expanded the methods section on the isolate selection. The use of a complete sampling of the invasive AMR isolates between 2006-12 and a minimum 10% random subsample of the susceptible isolates over the same time is emphasised now in the text (line 158-9). The overall sampled proportions of the total invasive isolates in any given year (Line 154-5) are highlighted as being between 20% and 34% for Klebsiella pneumoniae in any year between 2006-12.*

*When we tested and compared the diversity of the isolates observed in this study against the global and national population structures for both species, (described in previous work by some of the co-authors and by others (Holt et al., 2015; Moradigaravand et al., 2017; Chavda 2016)) we found that they reflected the diversity of either species found in other nosocomial settings, validating the dataset as representative of the expected range of diversity when studying these species in hospitalised patients (see Figs 2 and 3).*

*During the validation of the genetic and temporal (population dynamics) signals we tested for correlates after randomising the data and found the ST data associated with two year cycling of clones for Klebsiella only (shown in Figure S4), demonstrating the genetic population structures of the species were fundamentally different during this study. This, together with the decreased genetic (ST) variability among the AMR Klebsiella only (vs. non-AMR Klebsiella, AMR-Enterobacter and non-AMR Enterobacter), indicated that the dataset correctly contrasted the populations with respect to Klebsiella’s dependence on HGT or clonal spread for the dominant [cephalosporin] resistance vs. the ready emergence of the endogenous resistance in Enterobacter.*

2.	The authors used mostly short-read sequencing to characterize MGEs associated with AMR. Short-read sequencing is not ideal to characterize MGEs (although I completely understand the reasoning because of cost-limitations using long-read sequencing).

*We agree and used PacBio long read sequencing to define accurate consensus sequences from carefully selected representative genomes/MGEs, as presented in the manuscript to support our conclusions (see new figure S6). It’s true that we were not able to perform long read sequencing on all samples due to cost. But those selected PacBio sequences, combined with the short-read data, fully support our conclusions*

Some minor comments:

1.	There are confusion in the literature regarding the terms, clones, clades, clusters and lineages. The authors use lineage liberally but refer to different entities E.G. Line 83 lineage refers to different klebsiella spp and on line 42 it seems to refer to clones. Please be consistent.

*We have made alterations throughout to reflect the use of ‘lineage to refer to species and sub-species, and use the term clone to refer to ST’s.*

2.	Lines 74-80; why were the fluoroquinolones not mentioned. For enterobacter spp (less likely for klebsiella spp), this group of agents are considered as one of the 1st line treatment options for serious infections.

*We have included a sentence addressing the fluoroquinolones.*

3. Line 159; E. aerogenes has recently been re-classified as Klebsiella aerogenes.

*This was included and the relevant citation added.*

Reviewer #2: The authors present the results of a temporal analysis of MDR Klebsiella and Enterobacter from a single hospital site. The uniqueness of this study is that it allows a fine scale analysis in the fluctuations and population dynamics of these bacteria which are missed from the usual global collection studies, or even national studies which examine snapshots in time. From this they identify clear differences in population dynamics between the two species, suggesting Klebsiella fluctuates based on the cycling of globally disseminated clones carrying MDR plasmids, whilst Enterobacter is a stable population not affected by plasmid carriage and resulting clinically relevant resistance. This is largely driven by a chromosomal intrinsic ampC.

The data are well interpreted and presented, and I have a few comments below that the authors may wish to consider to provide more clarity to the paper

Major Points:

Line 137: Not really a major point, but there is a dramatic sub-selection of isolates for sequencing without any real description of why or how they were chosen. Cab we be assured they are representative of the population, especially since this is crucial in affirming that Klebsiella was more prone to circulating clones. On a similar line its not clear from the results text how in silico resistance prediction shows sensu stricto comprises the majority, especially given the clear mobility of resistance genes shown later.

*Please see our response to Reviewer 1 point 2.*

*We have amended the text at lines 164-5 to clarify the observation that sensu stricto represented the majority of MDR isolates.*

Line 151: It would be nice to see major lineages (STs) designated on the Kleb tree My main point revolves around the ESBL gene context. The section is excellently presented and makes the paper in my opinion. However i think some confirmation needs to be done. Is the observation of shared contexts because these islands co-locate on all instances of, for example, CTX-M-15? This could be checked against reference sequences on NCBI and also other long read collections such as the recent ST131 E. coli reports from some of this group. The suggestion on line 253 of shared networks of MGE is contingent upon these genes such as CTX-M-15 having the possibility of being able to be found in other contexts and needs to be confirmed. If CTX-M-15 can only be found in that single context it says nothing about sharing networks really, and only that CTX-M-15 always sits in this genetic context regardless of where it is found.

*Our interpretation refers to the shared gene pool among Enterobacteriaceae, where mosaic plasmids and/or their elements are frequently exchanged; we have now revised the relevant text sections to clarify this statement and added further references. To assess the conservation in large-scale datasets, we have now added figure S6 showing a comparison of the close environment of the main resistance cassettes, as analysed in figure 6, in a recently released, curated plasmid reference database (Brooks et al. 2019). These analyses show that whilst the elements can be found in the same conformation, there is considerable variation in the conservation of the CTX-M-15 associated gene content.*

Minor points:

Line 38: should read "success of specific lineages"

Line 45: should read "led to markedly"

Line 74: suggest rewording to "both K. pneumoniae and E. cloacae remain largely susceptible to other important groups of antimicrobials in the UK"

Line 92: remove "however"

Line 94-8: Sentence is far too long and has a poor grammatical flow. Same with line 104-8.

Line 99: Can you actually measure how antimicrobial resistance as competitive advantage in a hospital setting correlates with the population structure? Not sure it is done in this paper even if you can Line 111: Inclusion of "first" is confusing and doesnt make sense in this context. The 2.4 times more likely statement is questionable in the absence of detail of how the strains were isolated. I know this is in the methods but a succinct summary would benefit here. It needs to be clear if these are all clinical symptomatic isolates.

Line 375: please, just provide the method for assigning MLST here, paper hunts are not acceptable

*All minor points have been revised as suggested, and the MLST method cited.*

**Second round of review**

**Reviewer 1**

We will just have to agree to disagree on some om my major issues. The paper remains rather vague and the selection of the isolates that underwent WGS is biased towards AMR.

**Reviewer 2**

In the interests of fairness I have reviewed the revised manuscript solely on the basis of the comments I personally provided on the original manuscript.

I thank the authors for their measured response to my initial review, and am thankful that they have decided to take those comments on board in the spirit they were intended. I thank the authors for their clarification of the sub-selection performed for the genome sequencing. It is now more transparent, and whilst it is impractical to sequence every single isolate, readers are now fully informed of how decisions were taken to sequence which strains, and how this reflects the full spectrum of isolates obtained in the period.

I also thank the authors for the extra analysis (Fig S6) of the genetic context of ESBL genes. It does somewhat show my point that these ESBL genes are primarily in identical smaller MGEs nested within chromosomes or plasmids, and so some caveats need to be born in mind for looking at gene sharing networks for ESBL genes. However the extra figure does show quite considerable sequence variation in those ESBL MGEs in a well curated reference dataset and so the extra data does enhance the claim being made by the authors.

I also thank the authors for their attention to my minor edit comments.
